# Division of labor and growth during electrical cooperation in multicellular cable bacteria

**DOI:** 10.1073/pnas.1916244117

**Published:** 2020-02-24

**Authors:** Nicole M. J. Geerlings, Cheryl Karman, Stanislav Trashin, Karel S. As, Michiel V. M. Kienhuis, Silvia Hidalgo-Martinez, Diana Vasquez-Cardenas, Henricus T. S. Boschker, Karolien De Wael, Jack J. Middelburg, Lubos Polerecky, Filip J. R. Meysman

**Affiliations:** ^a^Department of Earth Sciences, Utrecht University, 3584 CB Utrecht, The Netherlands;; ^b^Department of Biology, University of Antwerp, B-2610 Wilrijk, Belgium;; ^c^Antwerp X-ray Analysis, Electrochemistry & Speciation (AXES) Research Group, Department of Chemistry, University of Antwerp, B-2020 Antwerp, Belgium;; ^d^Department of Biotechnology, Delft University of Technology, 2629 HZ Delft, The Netherlands

**Keywords:** cable bacteria, multicellularity, metabolism, nanoSIMS, stable isotope probing

## Abstract

Multicellularity is a key evolutionary innovation, leading to coordinated activity and resource sharing among cells, which generally occurs via the physical exchange of chemical compounds. However, filamentous cable bacteria display a unique metabolism in which redox transformations in distant cells are coupled via long-distance electron transport rather than an exchange of chemicals. This challenges our understanding of organismal functioning, as the link among electron transfer, metabolism, energy conservation, and filament growth in cable bacteria remains enigmatic. Here, we show that cells within individual filaments of cable bacteria display a remarkable dichotomy in biosynthesis that coincides with redox zonation. Nanoscale secondary ion mass spectrometry combined with ^13^C (bicarbonate and propionate) and ^15^N-ammonia isotope labeling reveals that cells performing sulfide oxidation in deeper anoxic horizons have a high assimilation rate, whereas cells performing oxygen reduction in the oxic zone show very little or no label uptake. Accordingly, oxygen reduction appears to merely function as a mechanism to quickly dispense of electrons with little to no energy conservation, while biosynthesis and growth are restricted to sulfide-respiring cells. Still, cells can immediately switch roles when redox conditions change, and show no differentiation, which suggests that the “community service” performed by the cells in the oxic zone is only temporary. Overall, our data reveal a division of labor and electrical cooperation among cells that has not been seen previously in multicellular organisms.

## Significance

Cable bacteria form centimeter-long, multicellular filaments whose energy metabolism involves cooperation among cells that separately perform oxidation of the electron donor and reduction of the electron acceptor. This cooperative division of labor is facilitated via long-range electrical currents that run from cell to cell along a network of conductive fibers. Here we show that biomass synthesis shows a surprising asymmetry along the filament: only the cells oxidizing the electron donor conserve energy for growth, while the other cells reduce electron acceptors without biosynthesis. Our study hence provides insights into the physiology of an unconventional chemolithotroph, which forms a multicellular electrically connected system with unique functional differentiation, integration, and coordination.

Cable bacteria belong to the candidate genera Electrothrix and Electronema within the *Desulfobulbaceae* family and are filamentous microorganisms that form up to centimeter-long chains of cells joined end to end by tight intercellular junctions (1–4). They inhabit marine and freshwater sediments (3, 5, 6) and have evolved a unique electrogenic metabolism that allows electron donors and acceptors to be harvested in widely separated locations. Cells within the same filament show a striking dichotomy in their metabolism, corresponding to a spatial zonation of redox half-reactions (1): cells located within deeper sediment layers perform the “anodic” oxidation of hydrogen sulfide (1/2H_2_S + 2H_2_O → 1/2SO_4_^2-^ + 4e^−^ + 5H^+^), while cells in the upper oxic layer of the sediment reduce oxygen (O_2_ + 4H^+^ + 4e^−^ → 2 H_2_O). Cable bacteria also have a high affinity for H_2_S (7), which allows them to efficiently scavenge H_2_S from the pore water, creating a centimeter-wide zone in the sediment where neither O_2_ nor H_2_S is detectable (1, 5, 8). Although there is no detectable H_2_S in this so-called suboxic zone, it has been shown that there is intense cryptic sulfur cycle operating, where cable bacteria immediately oxidize the H_2_S produced by sulfate reduction and FeS dissolution (7, 9). The spatial segregation of cathodic oxygen reduction in the oxic zone and anodic sulfide oxidation in the suboxic zone necessitates that electrons are passed from cell to cell over distances from micrometers to centimeters, and it was demonstrated that this electron transport indeed occurs inside the bacteria (10, 11). Cable bacterium filaments share a common periplasm containing a parallel network of fibers, and the fibers remain continuous across cell-cell junctions and stretch the entire length of a filament (4). Electrical measurements have recently shown that these periplasmic fibers are the conductive structures enabling long-distance electron transport (11). The remarkable electrical interaction between cells of the same filamentous organism provokes the question of how the electron flow is coupled to energy conservation, biosynthesis, and cell growth.

To examine this question, sediment cores with an active cable bacteria population were amended with either ^13^C-labeled bicarbonate (targeting inorganic C uptake) or propionate (targeting organic C uptake) in combination with ^15^N-labeled ammonium (proxy for general biomass synthesis). After 24 h of incubation, the redox zonation within the sediment was assessed by microsensor profiling. Subsequently, individual cable bacterium filaments were separately retrieved from the oxic (0 to 2 mm) and suboxic zones (5 to 10 mm) of the sediment, and their carbon and ammonium assimilation was determined using nanoscale secondary ion mass spectrometry (*Methods*).

## Results

### Tight Coupling Between Carbon and Ammonia Assimilation.

**Fig. 1.**
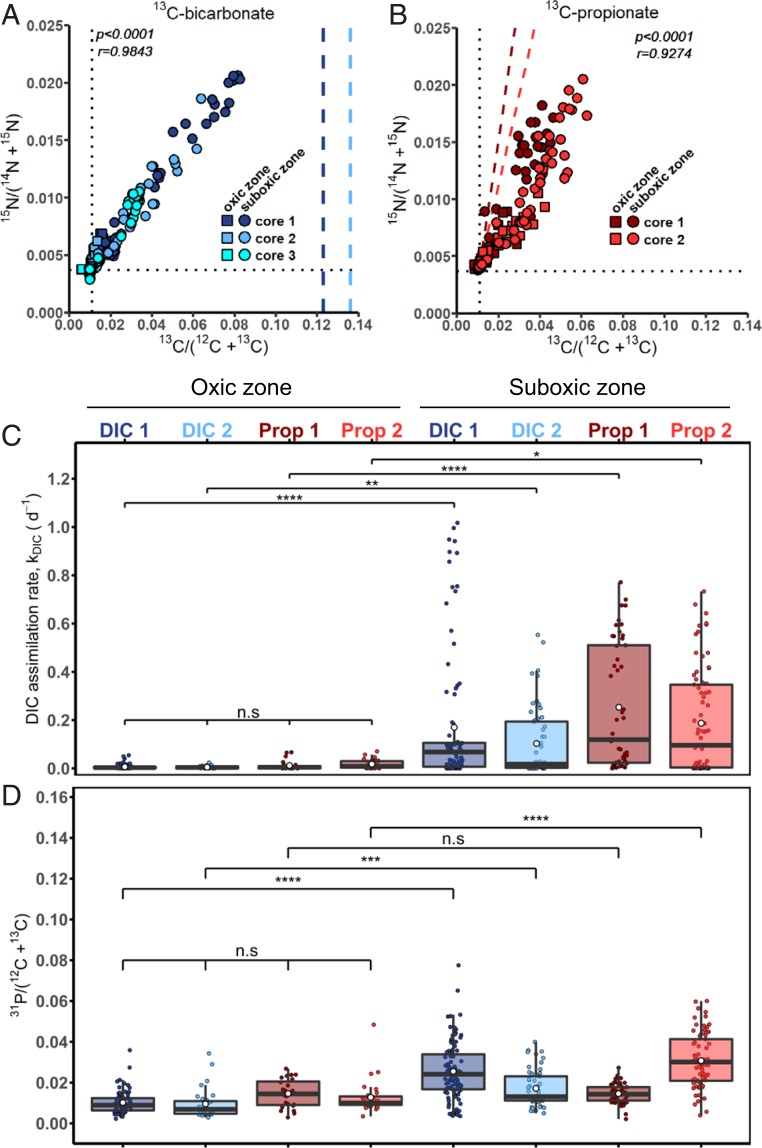
Assimilation of ^13^C- and ^15^N-labeled substrates by cable bacteria as measured by NanoSIMS. Shown are atom fractions in incubations with (*A*) ^13^C-bicarbonate (*n* = 347) and (*B*) ^13^C-propionate (*n* = 177). Each data point represents the mean ^13^C and ^15^N atom fraction for a segment (5 to 10 cells), or in some cases from multiple segments (16 to 65 cells), from an individual filament (see example images in *SI Appendix*, Fig. S1). Pearson correlation coefficient (*r*) and the corresponding *P* value are also shown. Colors and symbols differentiate between replicate sediment cores and redox zones in the sediment from which the filaments were retrieved, respectively. Dotted lines represent the natural ^13^C (0.011) and ^15^N (0.0037) atom fraction measured in filaments with no exposure to labeled substrates. Dashed lines in *A* show the ^13^C atom fraction in the DIC pool during the incubation. Dashed lines in *B* show the predicted ^13^C atom fraction in the filament segments due to assimilation of ^13^C-bicarbonate produced in the sediment by mineralization of the ^13^C-propionate by other community members (*SI Appendix*, *Methods*). (*C*) Boxplot of the inorganic carbon assimilation rates, k_DIC_ (d^−1^). Each data point represents the value calculated for the same segment, or multiple segments, as the ^13^C and ^15^N atom fractions data shown in *A* and *B*. Segments were grouped based on the redox zone (oxic vs. suboxic), treatment (^13^C-bicarbonate vs. ^13^C-propionate), and core replicates. For one ^13^C-bicarbonate incubation (cyan circles in Fig. 1*A*), assimilation rates could not be calculated due to the lack of porewater ^13^C-DIC data. For the ^13^C-propionate incubations, the bicarbonate assimilation rates were estimated from the ^15^N enrichments (*SI Appendix*, *Methods*), and the corresponding propionate assimilation rates are shown in *SI Appendix*, Fig. S2. White circles and horizontal lines show the mean and median assimilation rate, respectively. *P* values indicate significant differences between redox zones within the same replicate core; n.s., *P* ≥ 0.05 (not significant); *P < 0.05; **P < 0.01; ***P < 0.001; ****P < 0.0001. (*D*) Boxplot of the relative phosphorus content in cable bacterium filaments expressed as the ^31^P/C ion count ratio. Each data point represents the value calculated for the same segment, or multiple segments, as the ^13^C and ^15^N atom fractions data shown in *A* and *B*. Grouping of the segments, as well as the meaning of the *P* values and symbols used, is the same as in *C*. The values of the ^13^C and ^15^N atom fractions and the ^31^P/C ion count ratio’s for each segment used in this figure can be found in Dataset S1.

Examination of individual filaments (*n* = 524) revealed that ^13^C and ^15^N atom fractions were strongly correlated in both the ^13^C-bicarbonate (*P* < 10^−4^; *r* = 0.9842; *n* = 347) and ^13^C-propionate (*P* < 10^−4^; *r* = 0.9274; *n* = 177) incubations (Fig. 1 *A* and *B*). This strong correlation after a relatively short incubation period (24 h) suggests that the uptake of carbon and ammonia is tightly coupled in cable bacteria. Although directly comparable data are scarce, the coupling of the C and N uptake in cable bacteria seems to be different from that seen in other prokaryotes. For example, anoxygenic phototrophic sulfur bacteria *Chromatium okenii*, *Lamprocystis purpurea* (both unicellular), or *Chlorobium clathratiforme* (filamentous) showed no coupling between inorganic carbon and ammonia assimilation, and this was attributed to the accumulation of carbon in storage compounds (12). Similarly, C and N assimilation in diazotrophic cyanobacteria are also decoupled because of their ability to store fixed nitrogen as cyanophycin (13, 14) or because specialized cells are developed, such as N_2_-fixing heterocysts (15, 16). Cable bacteria do not show cell differentiation (4), but have been demonstrated to produce polyglucose inclusions (17). Apparently, polyglucose accumulation was either absent or insufficiently active during our experiments to distort the coupling of carbon and ammonia assimilation. Future studies should investigate whether a similar tight coupling between C and N assimilation is found in other prokaryotes that do not harbor C or N storage compounds or form specialized cells.

### Cable Bacteria Are Facultative Autotrophs.

Cable bacteria showed clear ^13^C enrichment in both the ^13^C-bicarbonate and ^13^C-propionate incubations. Thus, cable bacteria appear to be facultative chemoautotrophs (18), congruent with the presence of both the Wood-Ljungdahl pathway for CO_2_ fixation as well as the methylmalonyl-CoA pathway for propionate assimilation in their genome (17). Still, cellular ^13^C enrichment in the ^13^C-propionate incubation could be due to assimilation of ^13^C-bicarbonate produced through ^13^C-propionate mineralization by other members of the microbial community. The significant ^13^C enrichment of the dissolved inorganic carbon (DIC) pool measured at the end of the ^13^C-propionate incubation confirmed that substantial mineralization had occurred (excess atom fractions in the two replicate sediment cores reached *x*^E^[^13^C]_*DIC*_ *=* 0.0378 and 0.0715). We used the strong correlation between ^13^C and ^15^N atom fractions in the cable bacteria from the ^13^C-bicarbonate incubation (Fig. 1*A*) to account for the uptake of mineralized ^13^C-DIC, and thus, we could distinguish between bicarbonate and propionate uptake in the ^13^C-propionate incubation (*SI Appendix*, *Methods*). We found that the direct propionate uptake was only ∼7% of the inorganic C uptake (*SI Appendix*, Table S1 and Fig. S2; values are for filaments from the suboxic zone; *Discussion*). Hence, both autotrophic CO_2_ fixation and heterotrophic propionate assimilation were conjointly active in the cable bacteria population, but the autotrophic pathway clearly dominated biomass synthesis.

### Synchronous Assimilation of Carbon along the Filament in the Suboxic Zone.

**Fig. 2.**
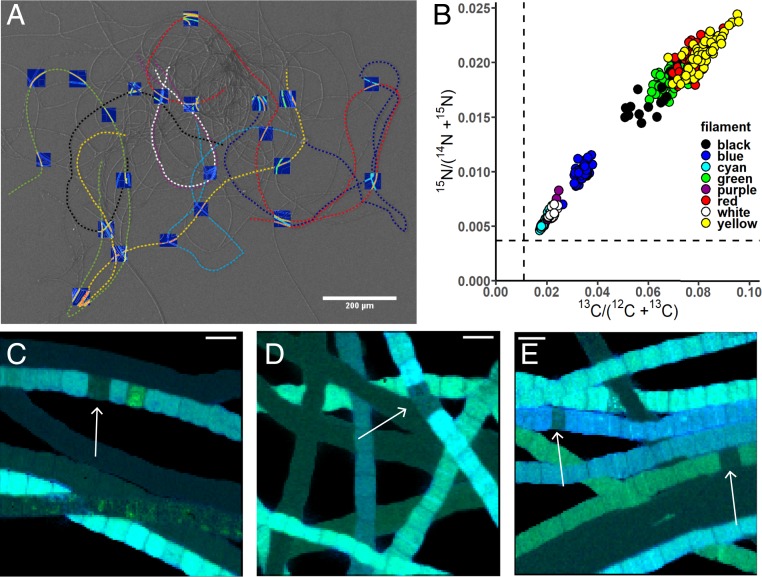
Variation in ^13^C and ^15^N atom fractions within filaments of cable bacteria. (*A*) Scanning electron microscopy (SEM) image of a bundle of cable bacterium filaments, with dashed colored lines indicating eight different filaments investigated in detail with NanoSIMS. Images of ^13^C atom fractions obtained by NanoSIMS are superimposed on the SEM image (the corresponding higher resolution image is shown in *SI Appendix*, Fig. S6). Filaments were retrieved from the suboxic zone of one of the cores incubated with ^13^C-bicarbonate. (Scale bar, 200 µm.) (*B*) Cross-plot of average ^13^C vs. ^15^N atom fractions in individual cells of the eight filaments analyzed. Dotted lines represent the natural ^13^C (0.011) and ^15^N (0.0037) atom fractions. The clustering of data points shows that intrafilament variation is substantially smaller than interfilament variation. The values of the ^13^C and ^15^N atom fractions for each of the cells can be found in Dataset S2. (*C*–*E*) Representative NanoSIMS images of the ^13^C and ^15^N atom fractions in cable bacteria from the (*C*) ^13^C-bicarbonate and (*D* and *E*) ^13^C-propionate incubation. Images are shown as overlays of the ^13^C (in green) and ^15^N (in blue) atom fractions. White arrows point to cells showing a decreased carbon uptake in an otherwise active filament. (Scale bars, 5 µm.) *SI Appendix*, Fig. S7 shows the original images of the ^13^C and ^15^N atom fractions, ^12^C^14^N secondary ion counts, and secondary electron images.

We examined individual cells from eight filaments (0.6 to 2.3 mm long) with a high carbon assimilation rate (k > 0.1 d^−1^; Fig. 2 *A* and *B*) that were retrieved from the ^13^C-bicarbonate incubation. This extensive analysis revealed limited variability in inorganic carbon assimilation within filaments (coefficient of variation of 9% to 19% among 16 to 65 cells within the same filament; *SI Appendix*, Table S2). Moreover, the inorganic carbon assimilation showed no systematic trend with distance along the filament (*SI Appendix*, Fig. S3). This remarkable homogeneity of C uptake within a filament is in stark contrast with the heterogeneity among filaments (coefficient of variation, 43% to 71%; *SI Appendix*, Table S1), thus suggesting that inorganic carbon assimilation is highly synchronous among cells within a cable bacterium filament. Although a similar homogeneous C uptake within filaments was reported for sulfide-oxidizing bacteria *Thioploca araucae* and *Thioploca chileae* (19), it is not typical for other filamentous prokaryotes. For example, filamentous cyanobacteria show heterogeneous C and N assimilation among cells within the same filament (13–16), due to differential formation of C and N storage compounds or because compounds with freshly fixed C and N are exchanged between differentiated cells (heterocysts vs. vegetative cells). High variability in C uptake between cells was also observed among cells within filaments of the heterotrophic bacterium *Candidatus Microthrix parvicella*. This variability was attributed to phenotypic heterogeneity and differential gene expression among cells, thus illustrating the colonial character of the organism (20).

### Electron Transport Takes Place across Inactive Cells.

Single cells were occasionally (∼0.15% of cells analyzed) encountered that showed significantly lower ^13^C and ^15^N enrichment compared with the remainder of the filament, suggesting metabolic inactivity (Fig. 2 *C*–*E*). Mechanical damage during filament retrieval and subsequent lysis of the labeled cell content are an unlikely explanation for this, as only single cells were affected and cell walls showed no signs of rupture. The presence of isolated inactive cells surrounded by highly active cells in both upstream and downstream directions indicates that electron transport can still take place across the filament. Therefore, a cell apparently does not need to be metabolically active to enable long-distance electron transport, as long as the conductive fiber network in the periplasm remains functional (11). This provides resilience to the electron transport and is highly beneficial for the functioning of the whole cable bacterium filament.

### Large Variation in Assimilation Rates among Filaments in the Suboxic Zone.

A sizeable fraction of filaments from the suboxic zone (110 of 269) showed high carbon assimilation rates (k = 0.1 to 1.02 d^−1^), whereas the remaining filaments were either inactive (82 of 269; k < 0.01 d^−1^) or minimally active (77 of 269; 0.01 d^−1^ < k < 0.1 d^−1^; *SI Appendix*, Figs. S1 and S4 and Table S1). The maximum assimilation rates observed here (k ∼ 1 d^−1^) are consistent with the doubling times for cable bacteria (∼20 h), as found in previous laboratory enrichment studies (2, 18). This variability in activity among filaments in the suboxic zone (coefficient of variation 55% to 86%; *SI Appendix*, Table S1) can be explained in several ways. One cause of variation could be oxygen availability, whereby the filaments that showed minimal or no activity had no access to oxygen during the 24-h incubation period. Differences in assimilation rates could also be the result of genetic diversity within the same phylogenetic population (12). Finally, the observed variation in assimilation rates could reflect differences in gene expression among individual filaments; for example, when filaments that belong to a different age/life cycle are in a different physiological state (12). Hence, it is possible that some filaments were younger and showed higher growth rates than other filaments. Overall, the observed variation in metabolic activity among filaments is striking, and future studies should better resolve the life cycle of cable bacteria to elucidate the cause of this variation.

### Anabolic Activity Is Highly Diminished in the Oxic Zone.

Our data show that filament label assimilation is strongly dependent on redox zonation (highly significant Kruskal-Wallis rank sum test, χ^2^ = 113.82; df = 7; *P* < 2.2·10^−16^; Fig. 1*C*). In contrast to filaments from the suboxic zone, all filaments in the oxic zone showed no or minimal label assimilation (*SI Appendix*, Fig. S1 and Table S1). Biomass synthesis thus appears to be strongly uncoupled from oxygen utilization. This observation concurs with recent genome analysis of cable bacteria, which reveals a complete absence of known membrane-bound terminal oxidases (17), thus leading to the hypothesis that oxygen reduction is entirely periplasmic and cannot be coupled to proton translocation and adenosine triphosphate (ATP) synthesis. The low but detectable label assimilation in a few filaments in the oxic zone may imply some limited basal maintenance metabolism, or alternatively, it could result from the migration of labeled filaments out of the suboxic zone and into the oxic zone during the 24-h incubation period.

The metabolic dependence with respect to redox zonation is also reflected in the phosphorus content. Filaments retrieved from the oxic zone had a significantly lower relative phosphorus content (quantified by the ^31^P/C ion count ratio) when compared with filaments from the suboxic zone (Kruskal-Wallis rank sum test, χ^2^ = 225.61; *P* < 2.2·10^−16^; Fig. 1*D*). We attribute this to a difference in the amount of intracellular polyphosphate inclusions between cells in the oxic and suboxic zones. Polyphosphate inclusions are long-chain polymers consisting of from 2 to 1,000 orthophosphate residues linked together by a high-energy phosphoanhydride bond that is similar to the bond in ATP (21). Cable bacteria have been shown to possess polyphosphate inclusions (17, 22, 23), although the size and density of these inclusions varied widely among filaments extracted from the same sediment sample (23). This is also reflected here by the large variability in the relative phosphorus content among filaments extracted from the same sediment core and redox zone (Fig. 1*D*). Polyphosphate can have many functions, and it has been thought to act as an energy storage system and to regulate the responses to stresses and adjustments for survival (21). The difference in the relative phosphorus content suggests a build-up of polyphosphate within the suboxic zone and breakdown of polyphosphate by cells residing in the oxic zone. Since biomass synthesis appears to be strongly uncoupled from oxygen reduction, cells in the oxic zone could use the polyphosphate inclusions as an energy reservoir or as a response to regulate oxidative stress.

### Cable Bacteria Have a High Capacity for Oxygen Reduction.

**Fig. 3.**
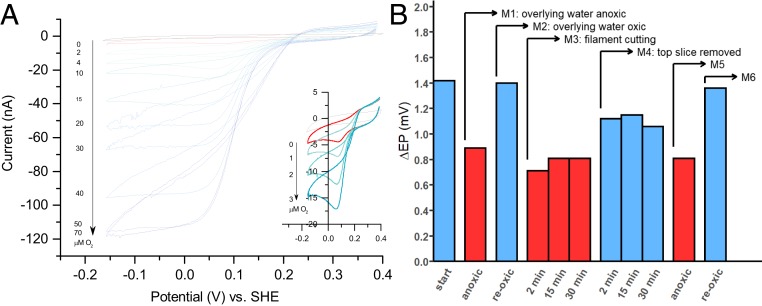
Oxygen reduction capability of cable bacteria and its dependence on redox zonation. (*A*) Cyclic voltammograms of intact cable bacteria deposited on a gold disk electrode (PBS at pH 7.4; scan rate, 0.02 Vs^−1^) at different concentrations of O_2_ (µM) in solution. (*Inset*) Cyclic voltammograms at low O_2_ concentrations. (*B*) Response of long-distance electron transport to a sequence of manipulations, as measured by the difference in EP (ΔEP) at the sediment-water interface and at 30 mm depth (the corresponding vertical profiles are show in *SI Appendix*, Fig. S5). Start, sediment with active long-distance electron transport by cable bacteria (high ΔEP); M1, anoxic overlying water (residual ΔEP due to diffusive electric potentials); M2, oxic overlying water; M3, sediment core was cut at a depth of 5 mm with a thin wire (3 mm below the oxic zone), but the sediment slice was left in place while the overlying water was oxic; M4, the top sediment slice was removed, so cells within the suboxic zone had instantly access to O_2_; M5, overlying water was made anoxic after 60 min (to verify that the response after M4 was due to cable bacteria); M6, oxic overlying water (to verify that the long-distance electron transport was reestablished).

To further elucidate the division of metabolic labor, we quantified the per cell capacity for oxygen reduction, using cyclic voltammetry (Fig. 3*A*). The observation of oxidation and reduction peaks (E^0^′ = 0.155 V vs. SHE) in the absence of O_2_ implies that redox-active sites are present on the outer surface of the cable bacteria. When O_2_ concentrations are gradually increased, the cathodic current increases, thus indicating that electrons are channelled onward to O_2_ (Fig. 3*A*). In the absence of cable bacteria, O_2_ does not affect the cathodic current. The cathodic current reaches a maximum of 1.1 × 10^−7^ A at 50 μM O_2_. Accounting for the total length of filaments on the electrode (12.03 mm, or 4,010 cells, as quantified by microscopy), this corresponds to an electron flow of 1.7 × 10^8^ electrons cell^−1^⋅s^−1^ and a cellular O_2_ reduction rate of 7.1 × 10^−17^ mol O_2_ cell^−1^⋅s^−1^. This rate should be considered as a conservative estimate, since some catalytic sites were likely not in contact with the electrode. This O_2_ reduction rate is 5 to 10 times higher than maximum rates obtained for unicellular aerobic sulfur oxidizers in continuous laboratory cultures (24, 25), and exceeds the typical in situ respiration rate of bacteria under nutrient-limiting conditions in aquatic sediments by 2 to 4 orders of magnitude (26). This sizeable capacity of cable bacteria for O_2_ reduction is not unexpected, as cable bacteria typically show similar volumetric filament densities in oxic and suboxic zones (2), and so a 10 to 20 times smaller number of “cathodic” O_2_-reducing cells in the shallow oxic zone (1 to 3 mm thick) must process all electrons originating from the many “anodic” H_2_S-oxidizing cells in the suboxic zone, which extends 10 to 60 mm deep into the sediment (5, 27).

### The Capacity for Oxygen Reduction Is Always Present.

Cells along a cable filament show a conspicuous redox dichotomy: they perform “anodic H_2_S oxidation” within the suboxic zone and “cathodic O_2_ reduction” within the oxic zone. To investigate whether cells are specialized, and hence whether the specific redox behavior is permanent, we recorded depth profiles of electric potential at various stages of a sediment manipulation experiment. The build-up of an electric potential with depth is a telltale sign of long-distance electron transport, thus signifying whether cable bacterium filaments are metabolically active (28, 29). The removal of the oxic top layer of the sediment was followed by a rapid recovery of the electric field (Fig. 3*B* and *SI Appendix*, Fig. S5). This demonstrates cells previously oxidizing sulfide rapidly switched to oxygen reduction once they had access to oxygen, and thus reestablished the long-distance electron transport. The response time (<2 min) was too quick to be mediated by gene expression. These data show not only that cable bacteria cells have an impressive enzymatic capacity for O_2_ reduction but also that this capacity appears to be continuously present in all cells. This “readiness” of cable bacteria cells to perform both redox half-reactions at any time could be beneficial in a dynamic environment, where redox zonation is frequently disturbed by physical alterations (e.g., bioturbation, wave action, tides). Alternatively, as cable bacteria are motile (30), having both the “cathodic” and “anodic” machinery operational at all times could be advantageous if parts of filaments would frequently relocate between oxic and suboxic zones.

## Discussion

Cable bacteria are presently the only filamentous microorganism known capable of long-distance electron transport (31). Long-distance electron transport allows an energetically favorable connection of sulfide oxidation to oxygen reduction, which gives cable bacteria a competitive advantage to flourish in the redox gradients that characterize aquatic sediments (31). A redox-stratified division of metabolic labor allows the multicellular cable bacteria to “mine” for hydrogen sulfide in deeper sediment layers, while still keeping contact with oxygen. Our results offer insights into this division of labor and growth, demonstrating that it is more complex and dynamic than previously thought.

### Oxygen Reduction Is Not Coupled to Anabolic Activity.

Our data paint an enigmatic picture of the oxygen utilization by cable bacteria. When cable bacteria are active within a sediment, geochemical evidence from both field and laboratory studies show that their activity is responsible for a large fraction (5% to 93%) of oxygen reduction within sediments (5, 8, 27, 28). Our voltammetry data confirm that cable bacteria have an impressive enzymatic capacity for O_2_ reduction (Fig. 3*A*). Moreover, when filaments have no access to oxygen, the electric field generated by cable bacteria rapidly disappears (Fig. 3*B*), confirming that oxygen (or nitrate) is necessary for the functioning of the organism (28). Still, this process of oxygen reduction is apparently not, or is only to a limited extent, coupled to energy conservation. Extensive analysis of individual filaments showed no to very little biosynthesis in cells from the oxic zone, in contrast to high levels of biosynthesis in cells from the suboxic zone (Fig. 1*C*). This is congruent with recent genomic analysis, which reveals no known proteins that can couple energy generation in cable bacteria to oxygen reduction (17). Hence, while cable bacteria are responsible for a large fraction of oxygen reduction in sediments, there appears to be no energetic gain involved for the cells that do so. The fact that biosynthesis only occurs within the suboxic zone is also congruent with the known O_2_ sensitivity of the Wood-Ljungdahl pathway that is used for inorganic carbon fixation (32).

### A Perspective on the Coupling Between Electron Transport and Energy Conservation inside Cable Bacterium Filaments.

**Fig. 4.**
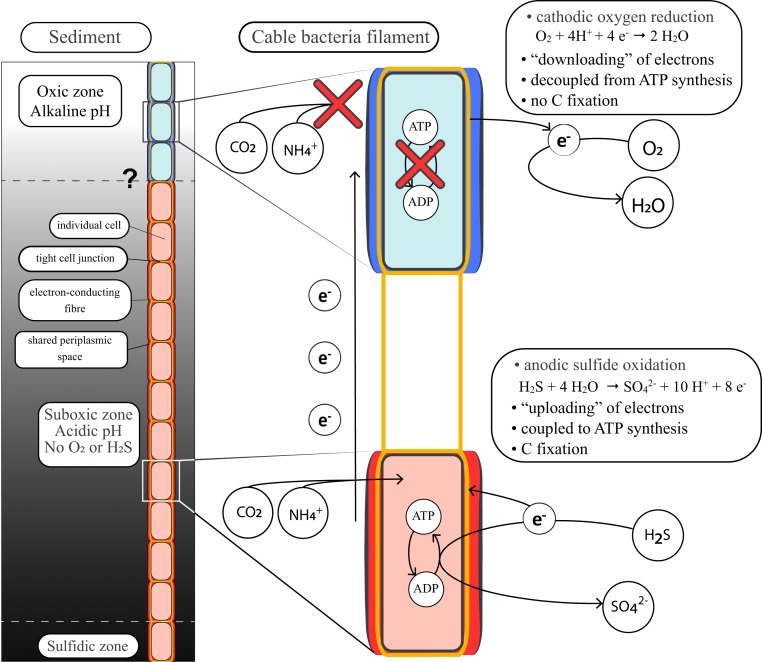
Schematic representation of the energy metabolism of a cable bacterium. The cells in the suboxic and sulfidic zone (red) perform anodic H_2_S oxidation. The free energy released by this redox half-reaction is utilized for generating ATP, thus allowing the assimilation of carbon and nitrogen, while the generated electrons are transported toward cells in the oxic zone via conductive fibers in the shared periplasmic space. Cells in the oxic zone (blue) utilize these electrons to perform cathodic O_2_ reduction, but this results in very little to no release of free energy and hence no capacity to assimilate carbon and ammonia.

The process of electron transport consists of three key steps (Fig. 4). First, electrons are generated by cells performing anodic sulfide oxidation, and this process can be coupled to energy conservation via substrate-level and oxidative phosphorylation through reversal of the canonical sulfate reduction pathway (17), similar to their close relative *Desulfurivibrio alkaliphilus* (33). The electrons produced by anodic sulfide oxidation are subsequently “uploaded” onto the conductive periplasmic fiber grid (11) and transported from cell to cell along the longitudinal axis of the filament to the oxic zone. The voltage drop along the periplasmic conductive structure of single filaments is ∼12 to 14 mV mm^−1^ (10), which implies a potential difference of only ∼40 µV across a single cell. Such a small potential difference would only generate 3.6 J per mole of electrons, which is three orders of magnitude lower than the minimum biological energy quantum needed to sustain life (34), and would require the passing by of >5,000 electrons to generate a single ATP molecule. Hence, electron transport across a cell is an unlikely source of free energy required for ATP synthesis. In the third step, electrons are “downloaded” from the periplasmic grid by cells performing cathodic oxygen reduction (31), and our results show that this step is not coupled to anabolic reactions. Accordingly, only the anodic sulfide-oxidizing half-reaction is coupled to energy conservation and allows for the observed biosynthesis and growth. The division of labor in terms of redox metabolism (cathodic and anodic cells cooperate) therefore generates highly asymmetric rewards in terms of cellular anabolism (only the anodic cells grow). To optimize the energy yield, we hypothesize that the redox potential on the periplasmic wire network in cable bacteria is closer to oxygen than to sulfide, so that most energy is recovered by the uploading of electrons from sulfide oxidation to the wire network, and little energy is dissipated by downloading of electrons from the wire network toward oxygen reduction. This hypothesis needs to be tested in further experiments.

### Division of Labor within Individual Filaments.

So why is there no apparent energy conservation during cathodic oxygen reduction? One proposition is that oxygen reduction in cable bacteria has been optimized for speed, rather than energy conservation (17). Cells in the oxic zone merely serve to reduce oxygen as fast as possible to efficiently evacuate electrons from the conductive wire network, thereby enabling the anodic cells in the deeper layers to use the periplasmic grid as an electron sink with a high redox potential. Although such asymmetric metabolism would benefit the filamentous organism as a whole, the oxic cells would bear the burden of the metabolic costs associated with oxygen reduction, which includes detoxification of reactive oxygen generated by high O_2_ reduction rates. Likely other metabolic costs are incurred in the oxic zone, as the high pH in the oxic zone could hamper the function of periplasmic and outer membrane proteins (17). Thus, the oxygen-respiring cathodic cells appear to provide a kind of “community service” to the filament by ensuring a relay of electrons to facilitate the growth of the sulfide-oxidizing anodic cells while they do not grow themselves.

Our results provide no indication that the cathodic cells receive a “reward” in return for their O_2_ reduction “service” to the anodic cells. One way to establish a mutually beneficial interaction would be an intercellular exchange of metabolites akin to that occurring between the heterocyst and vegetative cells in filamentous cyanobacteria (15, 16). However, this mechanism is unlikely in cable bacteria, given that their cells are separated by a rigid junction with no evidence of pores or channels (4). An alternative route for metabolite transfer could be the periplasm, which harbors the conductive fibers and is shared among all cells in the filament (10). However, a simple assessment of the length and time scales of diffusion (D = δ^2^/[2τ]) immediately reveals that such transport would be very slow. Given the diffusion coefficient of organic substrates in the periplasm (∼5 to 100 × 10^−12^ m^2^⋅s^−1^) (35), time scales required for diffusive transport along centimeter-long filaments (δ ∼ 1 cm) would amount to 5 to 100 d. Finally, any intercellular exchange of metabolites would necessarily imply a transfer of isotope label to the cells in the oxic zone, but no such carbon or nitrogen transfer was observed after a 24-h incubation period (Fig. 1).

This invokes the question of how cells in a cable bacterium filament collectively function. In order to perform long-distance electron transport, one part of the filament must be in contact with O_2_. One option is that cells in the oxic zone perform O_2_ reduction without biomass synthesis, but maintain a basal metabolism until all their energy reserves are exhausted and then die. They are then continuously replaced by new cells from the suboxic zone, which are pushed up as the filament in the suboxic zone grows. Alternatively, the community service in the oxic zone could have a temporary character. Cable bacteria show gliding motility (30), and microscopic evidence reveals they can instantaneously change their direction of motion (Movie S1). Filaments could move within the sedimentary redox gradients in such a way that different parts of the filament are in contact with O_2_ at different times. Individual cells thus could “take turns” in the oxic zone in a cooperative division of stress. Our phosphorus data are supportive to this idea. In the oxic zone, cells could temporarily rely on energy stores such as polyphosphates, and when cells subsequently retreat back into the suboxic zone, these energy reserves could be replenished. As such, one would expect polyphosphate reserves to be lower in cells in the oxic zone, as seen in our data (Fig. 1*D*). Our finding that cells have both the “cathodic” and “anodic” enzymatic machinery operational at all times (Fig. 3) is also congruent with the idea of a temporary community service in the oxic zone. It indicates that cable bacteria are geared up to cope with rapid redox changes, as would happen when filaments frequently relocate between oxic and suboxic zones.

Multicellularity has emerged independently more than 25 times in the history of life (36), and represents a highly diverse phenomenon, as it has been realized in different ways and with different degrees of integration (37, 38). Cable bacteria add another twist to this story, as the filaments display a unique form of metabolic integration facilitated by electrical currents. A key attribute of multicellularity is that differentiated cell types perform distinct functions, as this increases the fitness of the organism, even if such differentiation is costly to individual cells (36, 39). Cable bacteria fit this picture and display a remarkable division of labor: cells in the suboxic zone harvest energy from the oxidation of sulfide and assimilate inorganic carbon and ammonia, whereas cells in the oxic zone reduce oxygen without biosynthesis. While having access to the energetically favorable electron acceptor O_2_ increases the overall fitness of the organism, this comes at a cost of giving up biomass synthesis for the cathodic cells that perform the O_2_ reduction. Cable bacteria have evolved from a clade of strictly anaerobic organisms, and this may explain the absence of biosynthesis in the oxic zone. Only through functional differentiation can the filaments simultaneously carry out the functionally incompatible tasks of aerobic sulfide oxidation (strictly dependent on O_2_) and inorganic carbon fixation via the Wood-Ljungdahl pathway (inhibited by O_2_). A peculiar difference compared with other multicellular prokaryotes is that the division of labor between cells appears to be reversible. The external redox environment determines which functional cell mode is active, and cells appear capable of switching fast between modes when exposed to different redox conditions. This also explains why cells are not morphologically differentiated within a filaments.

## Materials and Methods

### Stable Isotope Probing.

Assimilation rates of carbon and ammonia by individual cable bacterium filaments were quantified by stable isotope probing combined with nano-scale secondary ion mass spectrometry (nanoSIMS). Parallel sediment cores with an active cable bacteria population were injected with a combination of ^13^C-bicarbonate (^13^C-DIC) and ^15^N-ammonia (^15^NH_4_^+^) to target assimilation of inorganic carbon and ammonia, and with a combination of ^13^C-propionate and ^15^NH_4_^+^ to target assimilation of organic carbon and ammonia. After 24 h of incubation, individual filaments were hand-picked from the oxic and suboxic zone of the sediment cores and analyzed by nanoSIMS. The nanoSIMS data were processed by Look@NanoSIMS (40) to determine the ^13^C-enrichment, ^15^N-enrichment, and relative phosphorus content (quantified as the ^31^P/[^12^C+^13^C] ion count ratio) of the individual cable bacteria. Additionally, porewater from the sediment cores was extracted and analyzed for the DIC and NH_4_^+^ concentrations and for the ^13^C-labeling of the DIC pool. Using modeling, these data were finally converted to the assimilation rates of inorganic and organic carbon.

### Cyclic Voltammetry.

The capacity of cable bacteria for oxygen reduction was quantified by cyclic voltammetry. A bundle of cable bacterium filaments was hand-picked from a sediment core with an active cable bacteria population and deposited onto a 1.6-mm BAsi gold disk electrode. Cyclic voltammetry was carried out in a PBS buffer (pH 7.4), using a conventional three-electrode electrochemical cell set-up (gold disks electrodes as working electrode, a glassy carbon rod as counter electrode, and a saturated calomel electrode immersed in a 20 mL PBS solution as reference electrode). Individual voltammograms were measured while increasing O_2_ concentrations in the solution in a step-wise manner from 0 to 70 µM.

### Sediment Manipulation Experiment and Electric Potential Measurements.

The capacity for oxygen reduction by cable bacteria cells performing anodic oxidation of sulfide was assessed by measuring electric potential (EP) in a sediment core with an active cable bacteria population. The sediment core was manipulated in steps that included variation in the oxygen availability in the overlying water column (oxic vs. anoxic) and sediment cutting. Each EP profile was measured in a different location.

Detailed description of the materials and methods is given in the *SI Appendix*.

### Data Availability Statement.

All data discussed in the paper are available in the *SI Appendix* and Datasets S1 and S2.

## Supplementary Material

Supplementary File

Supplementary File

Supplementary File

Supplementary File
