# Supplementary material for: Division of labor and growth during electrical cooperation in multicellular cable bacteria
Source: Proc Natl Acad Sci U S A. 2020 Feb 24;117(10):5478–85. doi: 10.1073/pnas.1916244117 (PMC7071850; doi:10.1073/pnas.1916244117)
Supplement: Supplementary File [file pnas.1916244117.sapp.pdf]

PNAS
www.pnas.org

Supplementary Information for

Division of labour and growth during electrical cooperation in multicellular cable bacteria

Nicole M. J. Geerlings[1*], Cheryl Karman[2,4], Stanislav Trashin[4], Karel S. As[1], Michiel V. M. Kienhuis[1], Silvia Hidalgo-Martinez[2,3], Diana Vasquez-Cardenas[2,3], Henricus T.S. Boschker[2,3], Karolien De Wael[4], Jack J. Middelburg[1], Lubos Polerecky[1*], Filip J. R. Meysman[2,3*]

N.M.J.Geerlings
Email: N.M.J.Geerlings@uu.nl

L. Polerecky
Email: L.Polerecky@uu.nl

F.J.R. Meysman
Email: filip.meysman@uantwerpen.be

This PDF file includes:

- Supplementary text
- Figures S1 to S8
- Tables S1 to S2
- Legend for Movie S1
- Legend for Dataset 1
- Legend for Dataset 2
- SI References

Other supplementary materials for this manuscript include the following:

- Movie S1
- Dataset 1
- Dataset 2

www.pnas.org/cgi/doi/10.1073/pnas.1916244117

Supplementary Information

Materials and Methods

Culturing conditions

Enrichment cultures were prepared from reduced, sulfidic sediment collected in the summer of 2016 from Rattekaai Salt Marsh (The Netherlands; 51.4391°N, 4.1697°E). This site was chosen because earlier studies documented the presence of cable bacteria in-situ (1, 2). The sediment was sieved (500 µm) to remove fauna, homogenized, and subsequently re-packed in polycarbonate cores (inner diameter 5.2 cm) as described before (1, 3). The sediment cores were submerged in artificial seawater (salinity of 32) and incubated in the dark for several weeks until an active cable bacteria population developed. The seawater was kept at 20 °C and bubbled with air to maintain 100% air saturation throughout the incubation.

Microsensor profiling

Microsensor profiling (O_2, H_2S, and pH) was performed to monitor the geochemical fingerprint and thus the developmental state of the cable bacteria population (4). The data were also used to discriminate between the oxic and suboxic zones in the sediment at the time of sampling. Microsensors were purchased from Unisense A/S (Denmark), connected to a four-channel Microsensor Multimeter (Unisense), and fixed in a two-dimensional micro-profiling system (Unisense) that enabled stepwise movement of the sensors. The software SensorTrace PRO (Unisense) was used to control the movement of the microsensors and log sensor signals. A general-purpose reference electrode (REF201 Red

Rod electrode; Radiometer Analytical, Denmark) was used as reference during the pH measurements.

Stable isotope probing experiments

Two separate incubation experiments were conducted to quantify the assimilation of carbon and ammonium by cable bacteria. The first took place in March 2017 and used sediment cores amended with ^{13}C-labeled bicarbonate (^{13}C-DIC) and ^{15}N-labeled ammonium (^{15}N-NH_4). The experiment was repeated in December 2017 and expanded by additionally incubating sediment cores amended with ^{13}C-labeled propionate and ^{15}N-NH_4.

Stock solutions used to amend the sediment cores were prepared as previously described (5). In March 2017, one stock solution was prepared by dissolving 62 mM ^{13}C-DIC (^{13}C atom fraction of 99%) and 0.35 mM ^{15}N-NH_4 (^{15}N atom fraction ≥98%) in artificial seawater. In December 2017, two stock solutions were prepared, one containing 62 mM ^{13}C-DIC and 0.40 mM ^{15}N-NH_4, and the other containing 11 mM ^{13}C-propionate (^{13}C atom fraction 99%, all C-atoms labelled) and 0.40 mM ^{15}N-NH_4. These concentrations were chosen because they were successful in labelling for nanoSIMS analysis in previous experiments (5). In both cases the artificial seawater contained no Mg and Ca ions to avoid precipitation of $Mg^{13}CO_3$ and $Ca^{13}CO_3$ as well as no bicarbonate and ammonium ions as to not dilute the label. The salts ($NaH^{13}CO_3$, $Na^{13}CH_3{}^{13}CH_2{}^{13}COO$ and $^{15}NH_4NO_3$) used for preparing the stock solutions were purchased from Sigma-Aldrich.

Labelling of the sediment cores was done by first inserting three sub-cores (inner diameter 1.2 cm) into the core without disturbing the sediment, and subsequently by injecting 500 µL of the labelled stock solution into each sub-core in ten 50 µL injections.

To ensure homogeneous spread of the label throughout the sediment, the syringe needle was inserted to a depth of 5 cm, and the liquid was released while slowly moving the needle upwards. The use of the sub-cores ensured that the label was spread within a well-constrained volume. One of the sub-cores was used to retrieve cable bacteria, while the other two were used for porewater analyses.

After label addition the cores were incubated for 24 h at 20°C to allow label assimilation by the cable bacteria. This incubation time was chosen because the doubling time was found to be around 20h (5, 6). In March 2017, this incubation was done in a dark container with no overlying water added on top of the sediment cores. The thin water film at the sediment surface was therefore in direct contact with air, which could have resulted in the dilution of the ^{13}C-DIC label close to the sediment-water interface due to CO_2 exchange. In December 2017, exchange with atmospheric CO_2 was avoided by performing the incubation in a sealed container filled with artificial seawater. For the cores amended with ^{13}C-DIC and ^{15}N-NH_4 the artificial seawater was labelled with ^{13}C-DIC and ^{15}N-NH_4 as well to ensure equal ^{13}C and ^{15}N labelling of the porewater and overlying water. This difference did not alter the results obtained from the March 2017 incubation.

Filament extraction

Clusters of cable bacterium filaments were picked under a microscope with fine glass hooks custom-made from Pasteur pipettes. Filaments were retrieved separately from the oxic (0-2 mm depth) and the middle of the suboxic (5-10 mm depth) zone of the sediment. The filaments were washed several times (> 3) in Milli-Q water (Millipore, The Netherlands) to eliminate precipitation of salt as described before (5), transferred onto

polycarbonate filters (pore size 0.2 µm; Isopore, Millipore, The Netherlands) pre-coated with a 5 nm thin gold layer, and air-dried in a desiccator for at least 24 h.

Scanning Electron Microscopy (SEM)

The polycarbonate filters were imaged with a scanning electron microscope (JEOL Neoscope II JCM-6000, Japan) to identify areas suitable for NanoSIMS analysis. This was done under a 0.1-0.3 mbar vacuum and a high accelerating voltage (15 kV) using a backscattered electron detector.

NanoSIMS analysis

Nano-scale secondary ion spectrometry (nanoSIMS) analysis was performed with the NanoSIMS 50L instrument (Cameca, France) to assess the assimilation of ^{13}C-bicarbonate or ^{13}C-propionate and ^{15}N-ammonium by individual cells of cable bacteria. Fields of view (FOV) selected through SEM were pre-sputtered with Cs^{+}-ions until secondary ion yields stabilized. Subsequently the primary Cs^{+}-ion beam (current: 1-2 pA, energy: 16 keV, spot size: 130 nm, dwell time: 1 ms/pixel) was scanned over the FOV (areas between 10×10 µm and 20×20 µm in size) while detecting secondary ions $^{12}C^{-}$, $^{13}C^{-}$, $^{12}C^{14}N^{-}$,$^{12}C^{15}N^{-}$ and $^{31}P^{-}$. To increase the overall signal the same FOV was imaged multiple times (10–200 frames), and the resulting ion count images were aligned and accumulated.

NanoSIMS data were processed using the Matlab-based software Look@NanoSIMS (7). After alignment and accumulation of the measured planes, regions of interest (ROIs), which corresponded to individual cable bacteria cells or segments of cable bacterium filaments (comprising between 5-10 cells), were drawn manually using

the $^{12}C^{14}N^{-}$ ion count image. The ROIs drawn around segments of cable bacteria were used to analyse differences between filaments extracted from different redox zones whereas the ROIs drawn around individual cells were used to analyse differences within cells belonging to the same filament. For each ROI, the ROI-specific ^{13}C atom fraction was calculated using the total $^{12}C^{-}$ and $^{13}C^{-}$ ion counts accumulated over all ROI pixels. Similarly, the ROI-specific ^{15}N atom fraction was calculated from the total $^{12}C^{14}N^{-}$ and $^{12}C^{15}N^{-}$ ion counts accumulated over all ROI pixels. ROIs were excluded from the final analysis if their ^{13}C or ^{15}N atom fraction varied significantly among measured planes.

The isotope data are presented as atom fractions, $x(^{13}C)$ and $x(^{15}N)$, or as excess atom fractions (also referred to as enrichment), $x^{E}(^{13}C) = x(^{13}C)_{s} - x(^{13}C)_{ref}$ and $x^{E}(^{15}N) = x(^{15}N)_{s} - x(^{15}N)_{ref}$, which is the difference between the atom fraction of the sample (s) relative to a reference value (8). Average ^{13}C and ^{15}N atom fractions of cable bacteria retrieved from a separate unlabelled sediment core were used as the reference values, i.e., $x(^{13}C)_{ref} = 0.011$ and $x(^{15}N)_{ref} = 0.0037$. The phosphorus content of the cable bacterium filaments is presented as the ion count ratio $^{31}P/(^{12}C+^{13}C)$ calculated from the total ion counts of P and C determined in the filament ROIs. This quantity should therefore be only interpreted as a relative measure of P that is comparable among cells and filaments analysed in this study.

Pore water analyses

The ^{13}C-labelling of the porewater dissolved inorganic carbon pool (DIC) was measured as previously described (9). Because of the limited porewater volume in the sampled sub-cores, these analyses could not be performed separately for the oxic and anoxic zones.

When possible, the handling was done under CO_2-free conditions (N_2 atmosphere) to minimize exchange with atmospheric CO_2. Under CO_2-free conditions, the top 3 cm of the sub-cores were sliced off and transferred into a 50 mL Greiner tube. The sediment was then centrifuged at 3000 rpm for 10 minutes. Again under anoxic conditions, the supernatant was retrieved and filtered over 0.45 µm pore size filters. Following filtration, 0.3 mL, 0.5 mL or 0.7 mL of the filtered porewater were injected into helium-flushed (5 min, flush rate of 70 mL min^{-1}) air-tight septum-capped vials (12 mL) that contained four drops of 85% H_3PO_4, which were subsequently analysed by GasBench IRMS. Only porewater from the experiment in December 2017 was measured. Therefore, assimilation rate constants could not be calculated from the experiment in March 2017. The porewater ammonium concentrations were determined by the colorimetric indophenol blue method (10). These values, in combination with the known amounts and labeling of the injected stock solution where then used to calculate the porewater ^{15}N-labeling based on mass balance.

Cyclic voltammetry on intact cable bacteria

Cyclic voltammetry was conducted in PBS pH 7.4 buffer using a PGSTAT 302 N potentiostat (Metrohm Autolab B.V., Utrecht, The Netherlands). A conventional three-electrode electrochemical cell was employed using BASi® gold disks electrodes (1.6 mm in diameter) as working electrode, a glassy carbon rod as counter electrode, and a saturated calomel electrode (SCE) immersed in a 20 mL PBS solution as reference electrode. The gold disk electrodes were sequentially polished with 3, 1 and 0.25 µm diamond and 0.05 µm alumina slurries, and electrochemically treated in 0.5 M H_2SO_4 by cyclic potential scans from 0.2 to 1.45 V versus SCE with a scan rate of 0.1 V s^{-1} until a characteristic

steady-state voltammogram was obtained. Further, the electrodes were incubated for 24 h in 8 mM mercaptohexanol (MH) dissolved in MilliQ water. Prior to use, the electrodes were washed with copious amount of MilliQ water. A clump of intact cable bacteria was collected from an independent sediment core enriched with an active cable bacteria population and deposited on the surface of the MH-modified gold electrode by the aid of a PBS buffer droplet. The oxygen concentration of the electrolyte solution was adjusted by injecting air-saturated PBS solution. Cyclic voltammograms were obtained at a scan rate of 20 mV s^{-1}.

To calculate the total length of the filament on the electrode (used for cyclic voltammetry measurements) bright field microscopy (Zeiss Axioplan 2, Germany) was used. The length of the filaments as well as the average cell length (3 μm) was determined by subsequent image analysis in ImageJ.

Sediment manipulation experiment

First, the presence of an active population of cable bacteria in the sediment core was confirmed by measuring vertical profiles of O_2, pH, H_2S and electric potential (EP) using microsensors (Fig. S5). Subsequently, the overlying water was purged for several minutes with N_2 gas to induce anoxia, and then purged with air for several minutes to re-establish oxic conditions. After each step, EP profiles were measured. Afterwards, the sediment was cut horizontally few mm below the oxic-suboxic boundary (5 mm depth) with a thin nylon thread (60 μm diameter), which disrupted the electron transport by the cable bacteria (11) and resulted in a decrease of the electric field in the sediment (12). EP depth profiles were

measured 2, 15 and 30 minutes after the cutting with the top 5 mm sediment layer left in place. Subsequently, the top sediment layer was removed, exposing previously suboxic sediment to an oxygenated water column, and depth-profiles of EP were measured after 2, 15 and 30 minutes. At the end, the overlying water was subjected to another cycle of induced anoxia and re-oxygenation while depth-profiling EP after each step.

Sediment cutting was done by fixing a ring with a height of 5 mm on top of the core liner and slowly pushing the sediment up with a plunger until the top of the sediment reached the top of the ring. Once the sediment was in place, a nylon thread was passed through the sediment guided by the slit between the core liner and the ring. Afterwards the slit was sealed with a water-proof adhesive tape to allow stable conditions during EP measurements. The sediment slice was removed by removing the adhesive tape around the ring and sliding the ring off the sediment core in one swift motion.

Electric potential (EP) was measured using an electric potential microelectrode (EPM) built at Aarhus University (Denmark) (13). The REF201 Red Rod electrode was used as a reference during the EP measurements. The EPM and the reference electrode were connected to a custom-made millivoltmeter with a resistance of $>10^{14}\ \Omega$.

Phase contrast microscopy

To observe the movement of cable bacteria with a phase contrast microscope special slides were constructed that allowed for a stable oxygen front. The slides were constructed by gluing slabs of microscope slide glass onto a microscope slide, creating a chamber in the centre of the slide (30 mm x 8 mm x 2 mm). Sediment was then placed in this chamber with a cover glass on top, and the narrow gap between the slide and cover glass was flooded

with artificial seawater to remove loose sediment particles. This created a clear division between sediment and glass, and cable bacteria could move into the space left between the chamber and edge of the cover glass (14). The movement of the bacteria within the chamber was analysed in a phase contrast microscope at 20°C. A ZEISS Observer Z1 (Zeiss, Göttingen, Germany) inverted microscope with a PALM automated stage and a 40x phase contrast objective was used.

Quantification of specific assimilation rates of C and N

Assimilation of carbon. Specific rates of carbon assimilation were estimated assuming that the cells assimilated carbon from two ^{13}C-labelled carbon sources, C1 and C2, and that for both carbon sources the increase in the cellular carbon content, *C*, followed first-order kinetics, $dC/dt = k_{C1}C + k_{C2}C$, where k_{C1} and k_{C2} denote the specific assimilation rate of carbon from the respective source. Both k_{C1} and k_{C2} represent the amount of C assimilated per unit time normalized to the C content of the cell, and are therefore expressed in units of d^{-1} (mol C (mol C)$^{-1}$ d^{-1}). These assumptions imply that the excess ^{13}C atom fraction of a cell, $x^E(^{13}C)$, changes in time according to the differential equation

$$\frac{dx^E(^{13}C)}{dt} = -k_{C1}\left[x^E(^{13}C) - x^E(^{13}C)_{C1}\right] - k_{C2}\left[x^E(^{13}C) - x^E(^{13}C)_{C2}\right]. \qquad (1)$$

where $x^E(^{13}C)_{C1}$ and $x^E(^{13}C)_{C2}$ denotes the excess ^{13}C atom fraction of the respective carbon source.

For the ^{13}C-bicarbonate incubation we assumed that the ^{13}C-enriched porewater DIC was the only carbon source, and that there was no uptake of propionate due to its low basal concentration in the porewater. Additionally, we assumed that the ^{13}C enrichment of

the DIC pool, $x^{E}(^{13}C)_{DIC}$, was constant during the incubation and equal to the value measured after the incubation. Based on these assumptions, the differential equation (1) is simplified to

$$\frac{dx^{E}(^{13}C)}{dt} = -k_{DIC}\left[x^{E}(^{13}C) - x^{E}(^{13}C)_{DIC}\right]. \quad (2)$$

The solution to this differential equation implies that the increase in the ^{13}C enrichment of the cell approaches the enrichment of the DIC in an inverted exponential fashion, i.e., evolves in time, t, as

$$x^{E}(^{13}C)_{t} = x^{E}(^{13}C)_{DIC} \times (1 - e^{-k_{DIC}t}). \quad (3)$$

Using this expression, the specific rate of inorganic carbon assimilation by a cable bacterial cell or a filament segment, k_{DIC}, was therefore calculated from its ^{13}C enrichment measured at the end of the ^{13}C-bicarbonate incubation as

$$k_{DIC} = -\frac{1}{t}ln\left(1 - \frac{x^{E}(^{13}C)_{t}}{x^{E}(^{13}C)_{DIC}}\right). \quad (4)$$

For the ^{13}C-propionate incubation, we assumed that the cable bacteria assimilated carbon from two sources: the added ^{13}C-enriched propionate as well as the porewater DIC. Since the added propionate far exceeded the propionate present in the porewater, the ^{13}C enrichment of the propionate, $x^{E}(^{13}C)_{P}$, was assumed to be constant during the incubation and equal to the value in the added stock (0.98). In contrast, the ^{13}C enrichment of the DIC pool, $x^{E}(^{13}C)_{DIC}$, was assumed to increase with time during the incubation due to the oxidation of the added ^{13}C-labeled propionate to CO_2 by the microbial community in the core. That this occurred was confirmed by the significant ^{13}C enrichment of the DIC pool measured at the end of incubation ($x^{E}(^{13}C)_{DIC}$ = 0.0378 and 0.0715 in the two replicate

sediment cores from which the cable bacteria were sampled). Because of this small ^{13}C enrichment of the DIC pool at the end of the incubation, we could approximate its increase during the incubation by a linear function of time, i.e.,

$$x^{\mathrm{E}}\left({}^{13}C\right)_{DIC,t} = x^{\mathrm{E}}\left({}^{13}C\right)_{P} k_{PO} t, \qquad (5)$$

where k_{PO} denotes the propionate oxidation rate constant. For the given ^{13}C enrichment of the added propionate ($x^{\mathrm{E}}\left({}^{13}C\right)_{P}$ = 0.98) and of the DIC after 1 day ($x^{\mathrm{E}}\left({}^{13}C\right)_{DIC,1d}$ = 0.0378 and 0.0715; see above), we calculated the propionate oxidation rate constants of 0.0386 d^{-1} and 0.073 d^{-1}. Taking into account these results, the differential equation (1) for the ^{13}C-propionate incubation is simplified to

$$\frac{dx^{\mathrm{E}}\left({}^{13}C\right)}{dt} = -k_{DIC}\left[x^{\mathrm{E}}\left({}^{13}C\right) - x^{\mathrm{E}}\left({}^{13}C\right)_{P} k_{PO} t\right] - k_{P}\left[x^{\mathrm{E}}\left({}^{13}C\right) - x^{\mathrm{E}}\left({}^{13}C\right)_{P}\right]. \qquad (6)$$

The solution to this differential equation implies that the ^{13}C enrichment of a cell assimilating carbon from these two sources will increase according to the function

$$x^{\mathrm{E}}\left({}^{13}C\right)_{t} = x^{\mathrm{E}}\left({}^{13}C\right)_{P} \times \left[\frac{k_P}{K}\left(1 - e^{-Kt}\right) + \frac{k_{PO}k_{DIC}}{K^2}\left(Kt - 1 + e^{-Kt}\right)\right], \qquad (7)$$

where $K = k_P + k_{DIC}$. How this expression was used to calculate the specific rate of propionate assimilation, k_P, is described below.

Assimilation of nitrogen. Specific rates of ammonium assimilation were estimated assuming that ammonium was the only nitrogen source, and that the increase in the cellular nitrogen content, N, followed first-order kinetics, $\mathrm{d}N/\mathrm{d}t = k_{\mathrm{NH4}}N$, where k_{NH4} denotes the specific assimilation rate of ammonium. This assumption implies that the excess ^{15}N atom fraction of a cell, $x^{\mathrm{E}}(^{15}\mathrm{N})$, changes in time according to the differential equation

$$\frac{dx^{\mathrm{E}}(^{15}N)}{dt} = -k_{\mathrm{NH4}}\left[x^{\mathrm{E}}(^{15}N) \quad - x^{\mathrm{E}}(^{15}N)_{NH_4}\right], \tag{8}$$

where $x^{\mathrm{E}}(^{15}\mathrm{N})_{NH4}$ is the excess ^{15}N atom fraction of the porewater ammonium.

For both incubations (^{13}C-bicarbonate and ^{13}C-propionate) we assumed that the ^{15}N enrichment of the porewater ammonium was constant during the incubation. Since we could not directly measure this enrichment, we estimated it based on the volume and ^{15}N labelling of the artificial seawater injected into the cores at the beginning of the incubation combined with the porewater NH_4 concentration and porewater volume determined after the incubation. Based on the assumption of constant $x^{\mathrm{E}}(^{15}N)_{NH_4}$, the solution to the differential equation (8) implies that the increase in the ^{15}N enrichment of the cell approaches the enrichment of the ammonium according to the function

$$x^{\mathrm{E}}(^{15}N)_t = x^{\mathrm{E}}(^{15}N)_{NH_4} \times (1 - e^{-k_{\mathrm{NH4}}t}). \tag{9}$$

Using this expression, the specific ammonium assimilation rate by a cable bacterial cell was therefore calculated from its ^{15}N enrichment measured at time *t* as

$$k_{\mathrm{NH4}} = -\frac{1}{t} ln\left(1 - \frac{x^{\mathrm{E}}(^{15}N)_t}{x^{\mathrm{E}}(^{15}N)_{NH_4}}\right). \tag{10}$$

Discrimination between the rates of inorganic carbon and propionate assimilation. The purpose of the incubation with the added ^{13}C-enriched propionate was to quantify the cell-specific propionate uptake rate, k_P. However, because the incubation with the ^{13}C-enriched DIC clearly showed that the cells assimilated inorganic carbon, and because there was a significant increase in the ^{13}C labelling of the DIC pool due to the conversion of ^{13}C-

propionate to ^{13}C-CO_2, the ^{13}C enrichment of cells from the ^{13}C-propionate incubation is described by a rather complicated function of time (see equation 7). Because this function depends on two unknown parameters, k_{DIC} and k_P, their values cannot be determined from the measurement of a cellular ^{13}C enrichment at a single time point. To allow separate discrimination of k_{DIC} and k_P, we took advantage of the fact that we simultaneously measured both the ^{13}C and ^{15}N enrichments in individual cable bacteria cells. Specifically, we exploited the observation from the ^{13}C-bicarbonate incubation that the cellular assimilation of ^{13}C-DIC and ^{15}N-ammonium assimilation were strongly coupled (correlation coefficient $r=0.9843$, $p<0.001$), i.e., the ratio between the rate constants k_{DIC} (see equation 4) and k_{NH4} (see equation 10) had a well-defined fixed value (denoted here as $r_{C/N} = k_{DIC} / k_{NH4}$).

Overall, we assumed that the coupling between the assimilation of DIC and NH_4 was the same during both the ^{13}C-bicarbonate and ^{13}C-propionate incubations. Thus, for a given ^{15}N enrichment of a cell from the ^{13}C-propionate incubation, we first determined its specific ammonium assimilation rate constant k_{NH4} from equation (10). Then we used the $r_{C/N}$ factor determined from the results of the ^{13}C-bicarbonate incubation and predicted the corresponding rate constant of inorganic carbon assimilation as $k_{DIC} = r_{C/N} \times k_{NH4}$. Using this rate constant, we then found k_P such that the ^{13}C enrichment predicted by equation (7) was equal to the measured value. Since an analytical solution to equation (7) for k_P does not exist, we found this solution numerically.

Statistical Analysis

In the isotope labelling experiments, there were four biological replicates (cores) and each core had two categorical treatments (oxic and suboxic), providing eight samples in total. All samples showed a non-normal distribution and unequal variances, while sample sizes were also variable (n between 24 and 94, Table S1). To evaluate the effect of the different treatments, a non-parametric Kruskal-Wallis test was performed, followed by a Dunn's post-hoc test to assess which sample pairs were statistically different. The R package "dunn.test" was used for pairwise comparisons. To avoid family-wise error rates, while retaining sufficient statistical power, the Holm-Bonferroni p-adjustment was used (15).

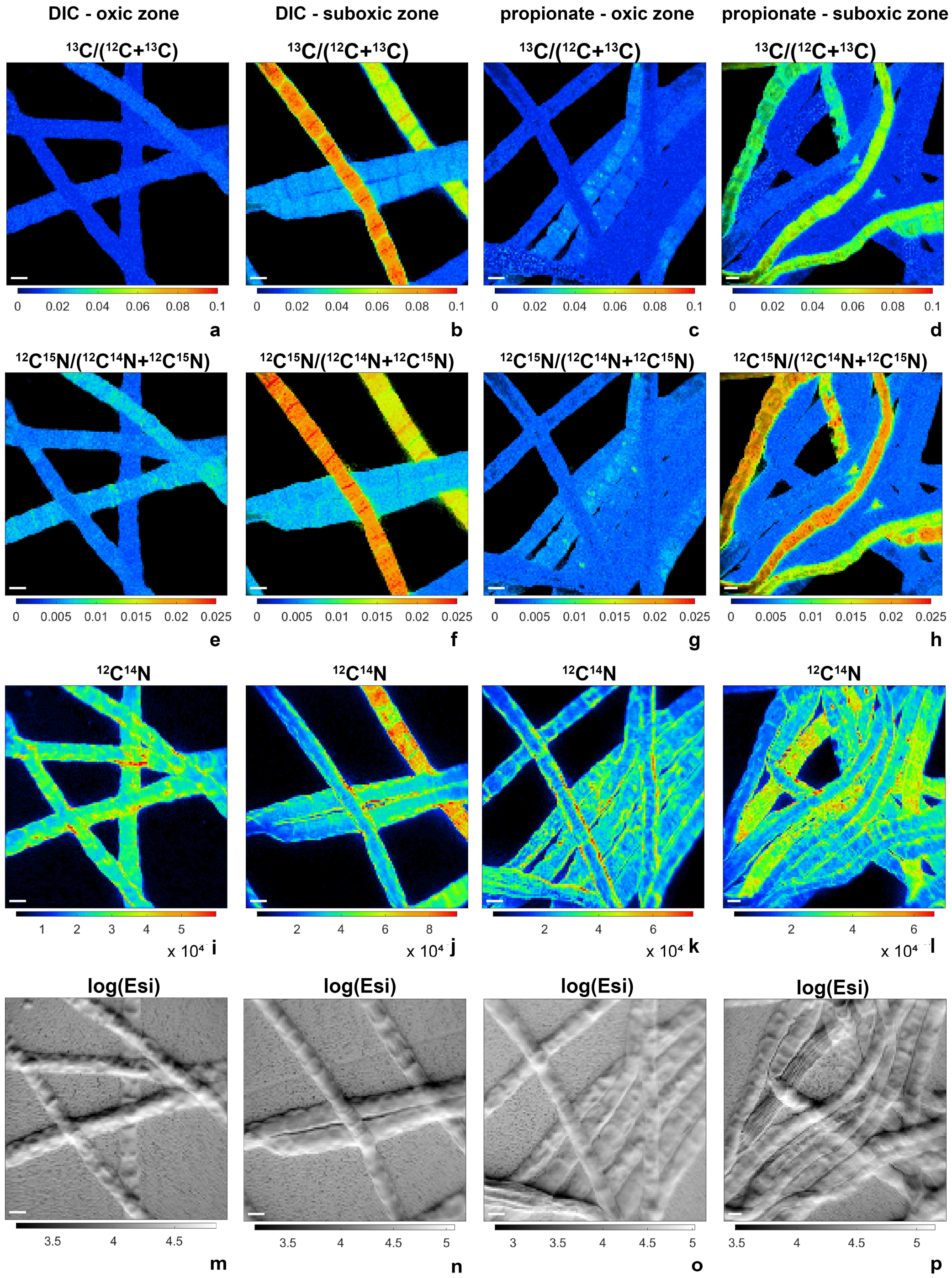

Fig. S1. Images of ^{13}C and ^{15}N assimilation by cable bacteria. Representative images of the ^{13}C atom fraction (a-d), ^{15}N atom fraction (e-h), $^{12}C^{14}N^-$ ion counts (i-l) and secondary electrons (m-p) in filaments from the different incubations and redox zones: filaments incubated with ^{13}C-bicarbonate, and retrieved from the oxic zone (a, e, i, m) and from the suboxic zone (b, f, j, n); filaments incubated with ^{13}C-propionate, and retrieved from the oxic zone (c, g, k, o) and from the suboxic zone (d, h, l, p). Scale bars are 3 µm. A filament segment was defined as a region of interest (ROI). The counts of secondary ions $^{12}C^-$, $^{13}C^-$, $^{12}C^{14}N^-$ and $^{12}C^{15}N^-$ were accumulated over the ROI pixels to calculate the average ^{13}C and ^{15}N atom fractions per filament.

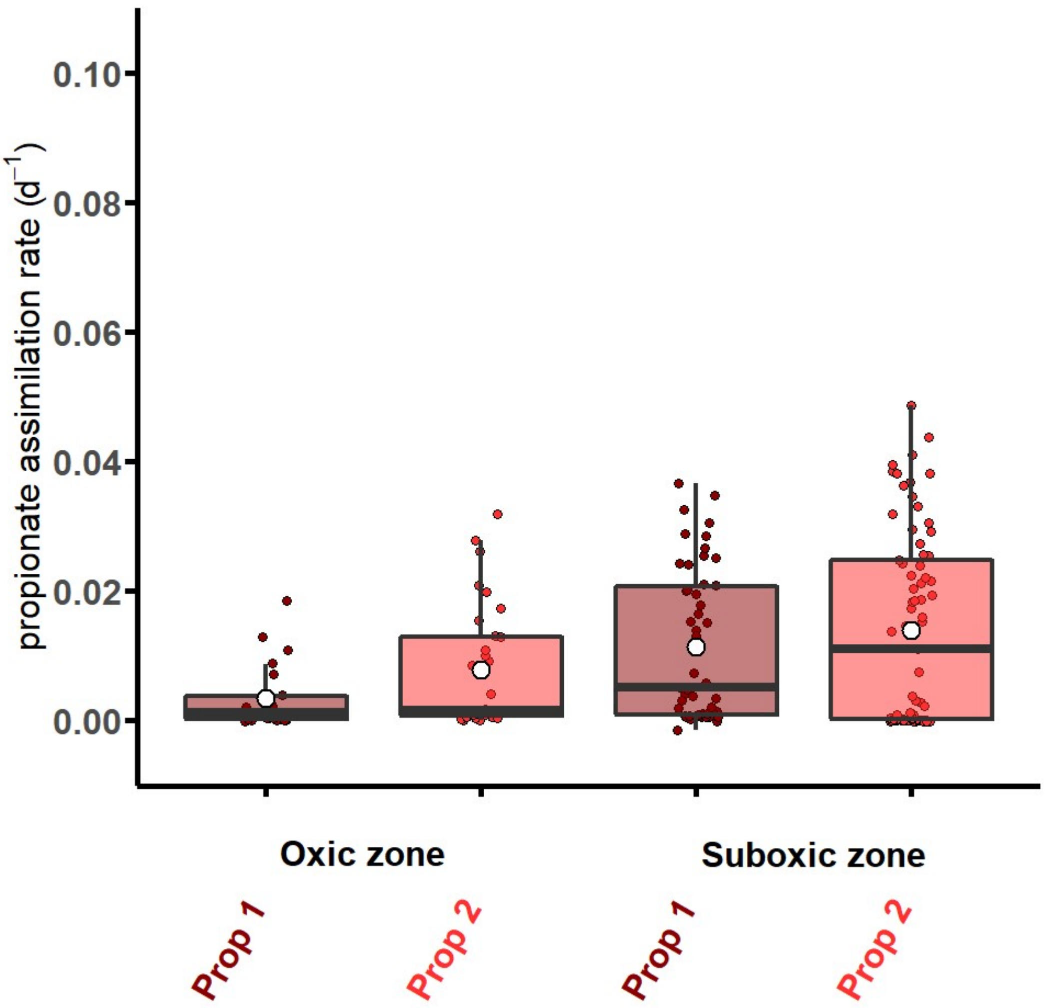

Fig. S2. Boxplot of propionate assimilation rates by cable bacteria. Shown are rates by filament segments retrieved from the oxic and suboxic zone of two replicate sediment cores from the ^{13}C-propionate incubation. The rates were calculated from the measured ^{13}C and ^{15}N atom fractions as described in the Methods. White circles and horizontal bars correspond to the mean and median assimilation rates, respectively, boxes extend from 0.25 to 0.75 quantile.

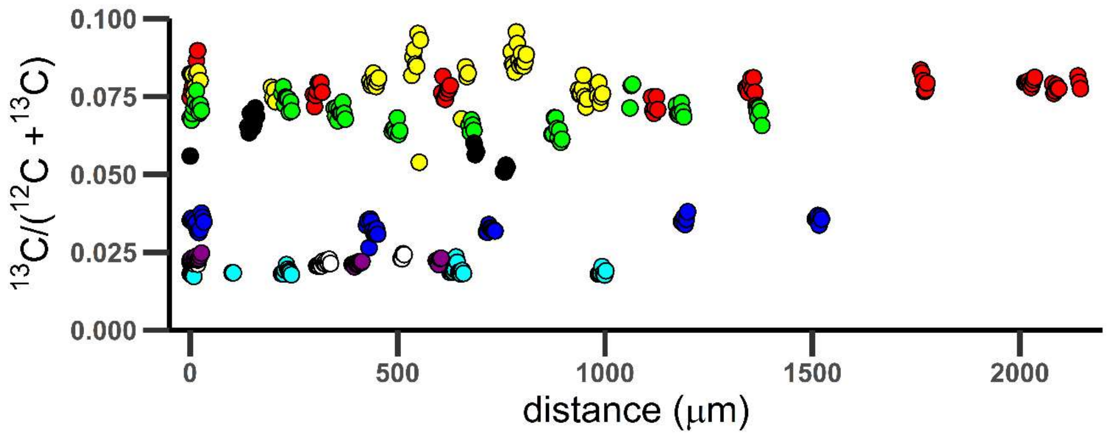

Fig. S3.: The ^{13}C atom fraction of cells along the length of the filament. Each data-point represents the average ^{13}C atom fraction for one cell along a filament. The distance between cells along the filament was determined from the SEM image shown in Fig. 2a. The colours correspond to the filaments as defined in **Fig. 2a-b**.

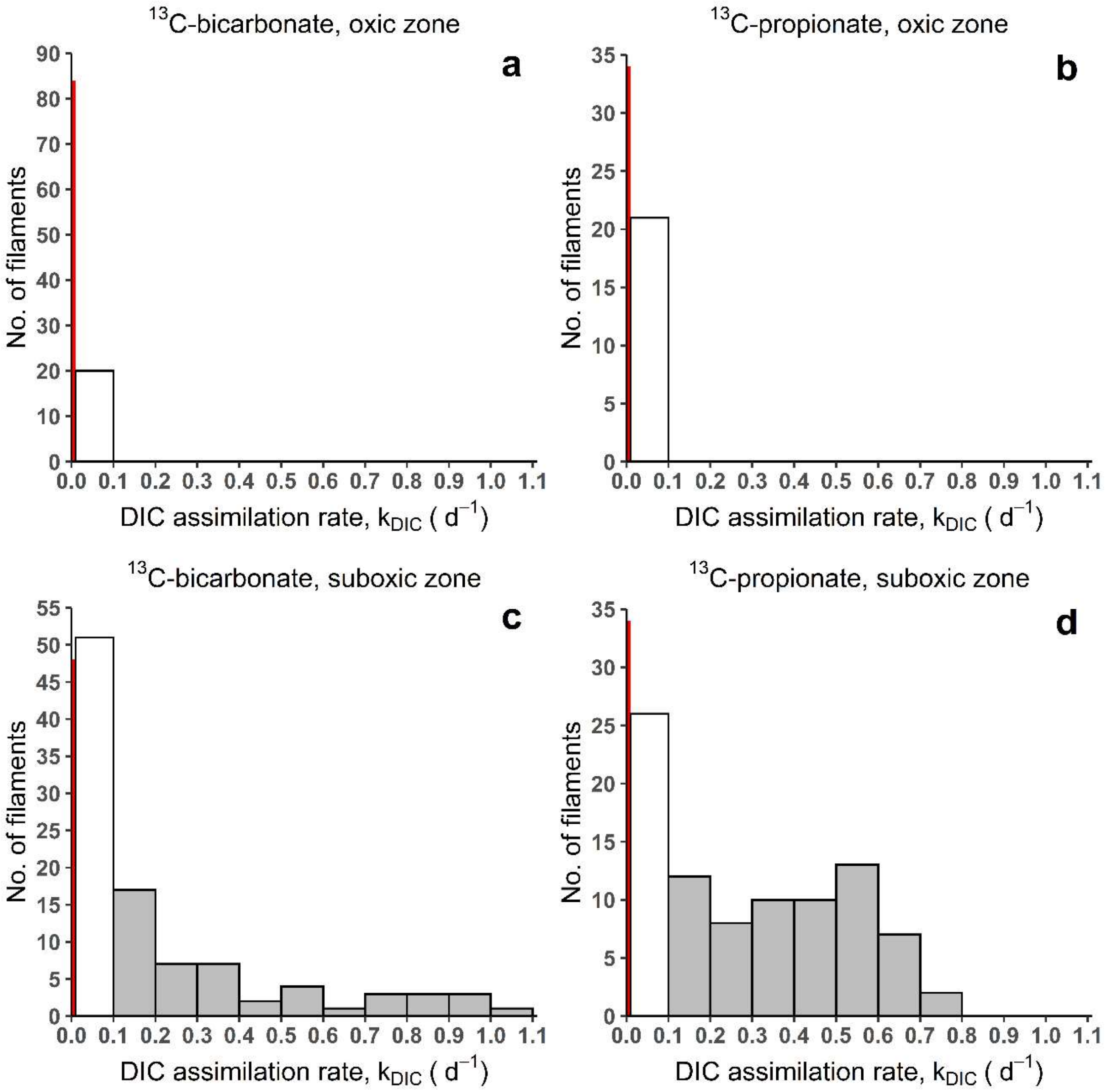

Fig. S4. Histograms of inorganic carbon assimilation rates by cable bacteria. Histograms are shown separately for filaments retrieved from the oxic and suboxic zone of sediment cores incubated with ^{13}C-bicarbonate and ^{13}C-propionate. For all histograms the first bin (0-0.01 d^{-1}, red) includes filaments classified as 'inactive' based on the fact that their ^{13}C enrichment was not significantly different from the filaments with no exposure to labelled substrates (control filaments). The second bin (0.01-0.1 d^{-1}, white) includes filaments classified as 'minimally active'. Filaments with the assimilation rates >0.1 d^{-1} (grey) were classified as 'active'.

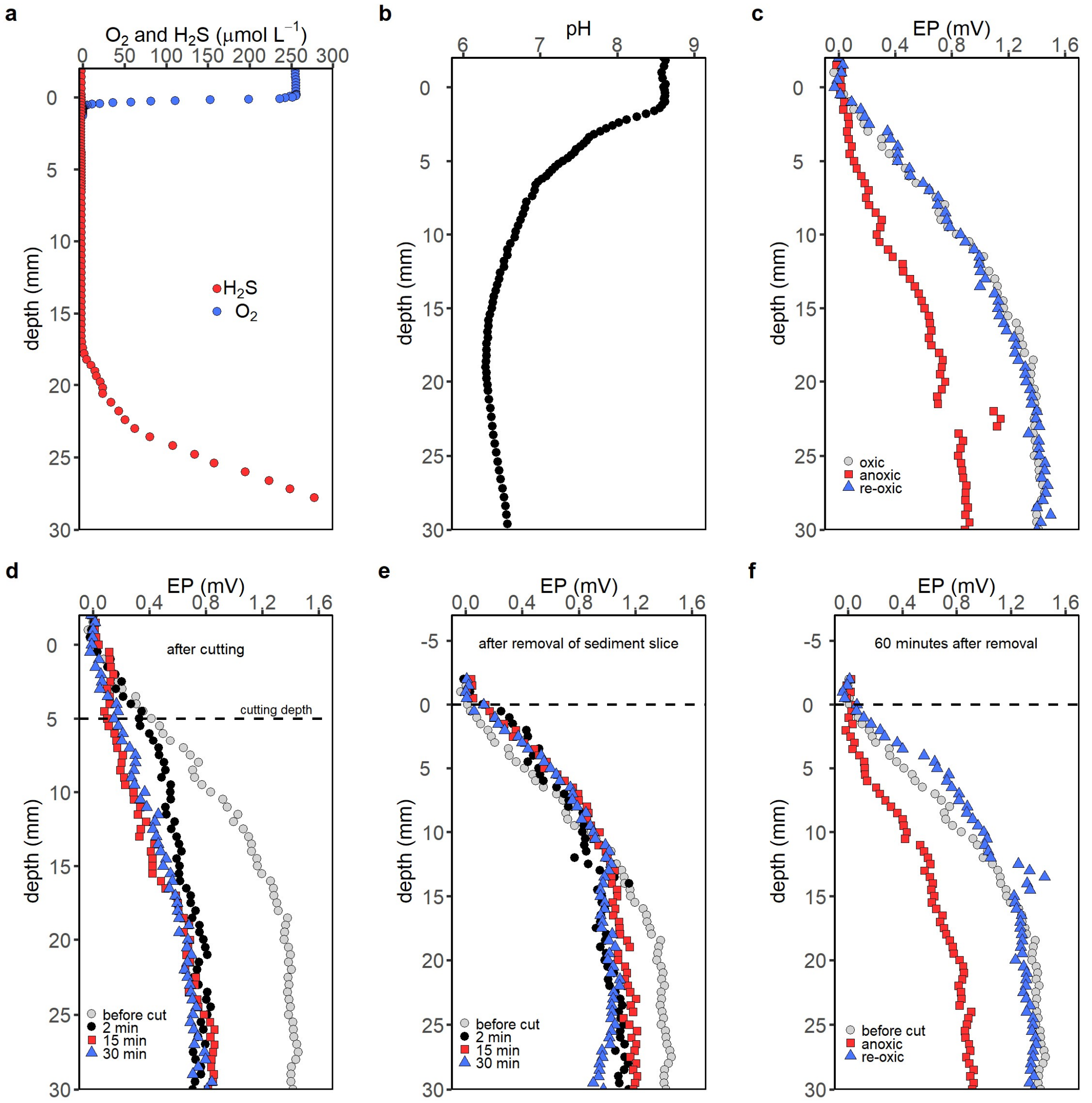

Fig. S5. Vertical profiles of O_2, H_2S, pH, and the electric potential (EP) in a sediment core with active cable bacteria during the sediment manipulation experiment. (a) Oxygen and sulfide depth profiles, **(b)** pH depth profiles. The combination of these profiles showed the presence of an active cable bacteria population. The oxygen depth profile was used to determine the oxygen penetration depth. The EP data were used to calculate the difference in electric potential (ΔEP) shown in Fig. 3. **(c)** EP profiles were measured in an intact core with the conditions in the overlying water sequentially altered from oxic (light grey) to anoxic (red) and back to oxic (blue). Each profile corresponds to an average of two replicate profiles. **(d)** Sediment cutting experiment: EP profiles were measured 2 min (black), 15 min (red) and 30 min (blue) after the sediment was cut horizontally with a thin wire at a depth of 5 mm. Each profile is a single measurement. **(e)** Removal of the cut sediment layer: EP profiles were measured 2 min (black), 15 min (red) and 30 min (blue) after the top 5 mm of sediment was removed and O_2 became available to the cable bacteria close to the new sediment-water interface (indicated by dashed line). Each profile is a single measurement. **(f)** EP profiles were measured 60 min after the top 5 mm of sediment was removed and the conditions in the overlying water were sequentially altered from anoxic (red) to oxic (blue) conditions. Each EP profile corresponds to an average of two replicate profiles. In panels (**d, e, f**) the original EP profile (light gray) is included for comparison. When necessary the profile was shifted downwards to reflect the correct position of the sediment-water interface.

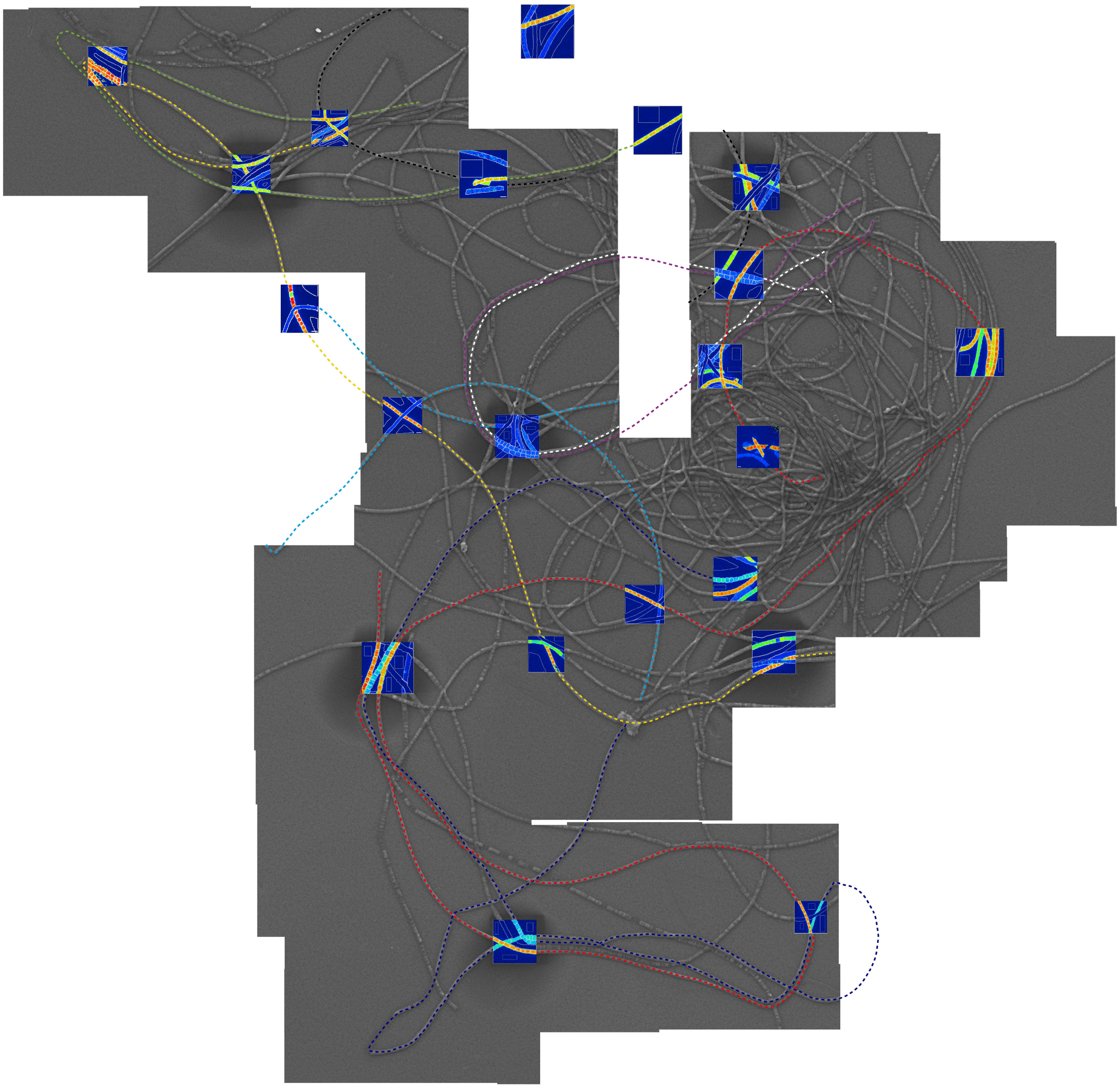

Fig. S6. Stitched Scanning Electron Microscopy (SEM) images of a bundle of cable bacterium filaments with a higher resolution that the one shown in **Fig. 2a**. Dashed coloured lines indicate some of the followed filaments investigated in detail with NanoSIMS. Images of ^{13}C atom fractions obtained by NanoSIMS are superimposed on the stitched SEM image.

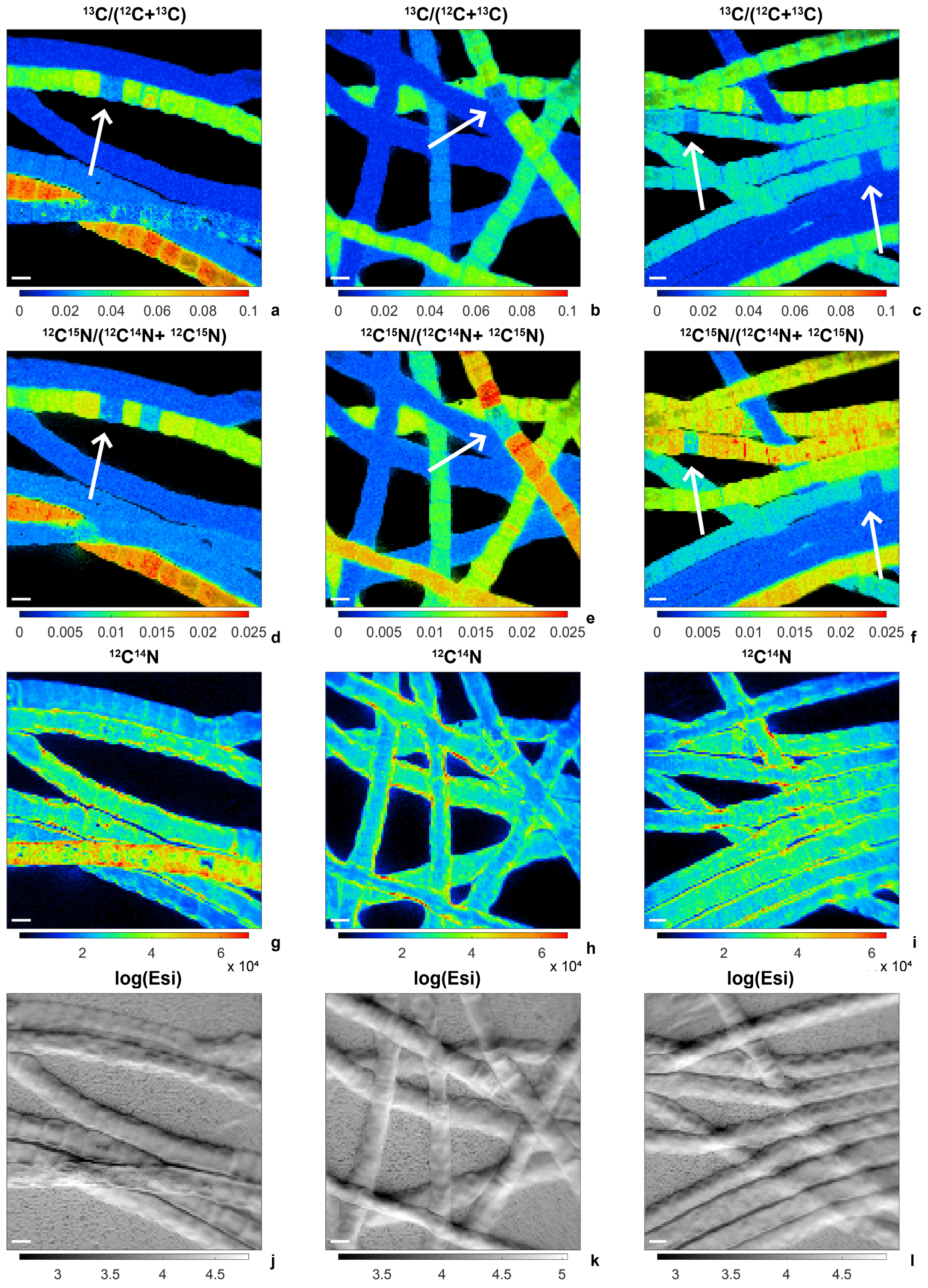

Fig. S7: **NanoSIMS images of the ^{13}C atom fraction (a-c), ^{15}N atom fraction (d-f), $^{12}C^{14}N^{-}$ ion count (g-i), and secondary electrons (j-l) in cable bacteria** from the (**a, d, g, j**) ^{13}C-bicarbonate and (**b-c, e-f, h-i, k-l**) ^{13}C-propionate incubation. White arrows point to cells showing decreased carbon and nitrogen assimilation in an otherwise active filament. The images correspond to the rgb overlay images shown Fig. 2c-e. Scale bars are 3 µm.

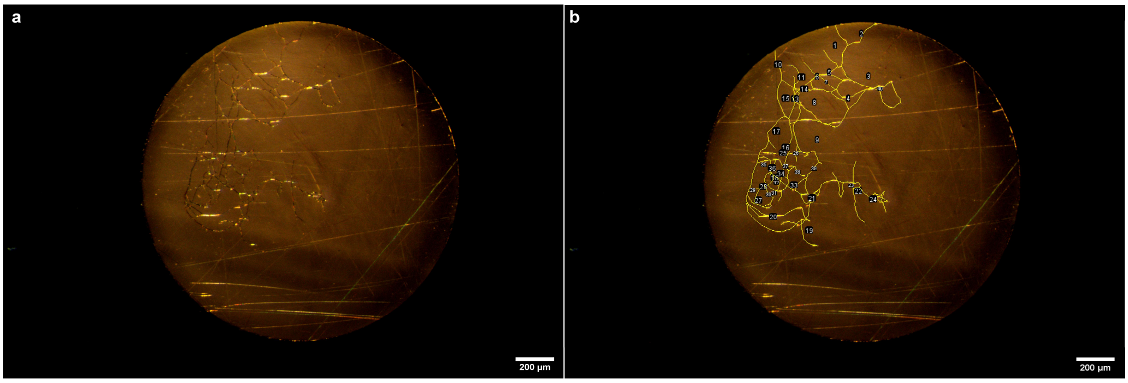

Fig. S8: (a) Bright field microscopy image of the cable bacteria used for cyclic voltammetry measurements and the (b) overlying lines drawn in ImageJ to determine the total length of the filaments.

Table S1. Variability of carbon assimilation rates among individual filaments of cable bacteria. Shown are the total number of filaments measured per replicate sediment core, and the corresponding amounts and percentages of filaments classified as 'inactive' (k_{DIC}=0-0.01 d^{-1}), 'minimally active' (k_{DIC}=0.01-0.1 d^{-1}), and 'active' (k_{DIC}>0.1 d^{-1}). For both incubations (^{13}C-bicarbonate and ^{13}C-propionate) most of the filaments retrieved from the oxic zone were inactive and only a small percentage was minimally active. Activity of the filaments from the suboxic zone was higher but still a large fraction of the filaments showed no or only a minimal activity. The average inorganic carbon assimilation rates are given separately for the active and minimally active filaments from both treatments. The standard deviation and the corresponding coefficient of variation illustrate considerable variability among individual filaments within the active and minimally active sub-populations. The ratio between the assimilation rates of propionate and inorganic carbon, k_P/k_{DIC}, shows that for filaments from the suboxic zone of the sediment cores labelled with ^{13}C-propionate only a small part (~7%) of the assimilated ^{13}C originates directly from the ^{13}C-propionate, whereas most of it originates from the ^{13}C-DIC produced through ^{13}C-propionate remineralization (see Methods). In contrast, for filaments from the oxic zone the contribution of ^{13}C-propionate to the total carbon uptake is much larger (22-36%, calculated as $k_P/(k_P+k_{DIC})$).

	^{13}C-bicarbonate				^{13}C-propionate			
	Oxic zone		Suboxic zone		Oxic zone		Suboxic zone	
	Core 1	Core 2	Core 1	Core 2	Core 1	Core 2	Core 1	Core 2
Total # filaments	73	31	94	53	24	31	49	73
Inactive	59 (81%)	25 (81%)	26 (28%)	22 (41%)	18 (75%)	16 (52%)	7 (14%)	27 (37%)
Min. active	14 (19%)	6 (19%)	39 (41%)	12 (23%)	6 (25%)	15 (48%)	16 (33%)	10 (14%)
Active	0 (0%)	0 (0%)	29 (31%)	19 (36%)	0 (0%)	0 (0%)	26 (53%)	36 (49%)
k_{DIC} active (d^{-1})	-	-	0.48 ± 0.34	0.27 ± 0.13	-	-	0.45 ± 0.19	0.37 ± 0.17
Coefficient of variation	-	-	71%	48%	-	-	43%	47%
k_{DIC} min. active (d^{-1})	0.024 ± 0.014	0.015 ± 0.005	0.054 ± 0.030	0.029 ± 0.025	0.040 ± 0.024	0.033 ± 0.016	0.040 ± 0.022	0.040 ± 0.034
Coefficient of variation	61%	31%	55%	85%	61%	49%	57%	86%
k_P/k_{DIC}	-	-	-	-	0.561±0.266	0.283±0.306	0.067±0.077	0.069±0.051
^{13}C-DIC ratio of the porewater	0.123	0.136	0.123	0.136	0.0484	0.0821	0.0484	0.0821

Table S2. Variability of carbon assimilation rates among cells within individual filaments of cable bacteria from the suboxic zone. Shown are statistics for the eight individual filaments retrieved from the suboxic zone from the bicarbonate incubation marked with different colours in **Fig. 2a**, including the distance (in mm and in number of cells) between the first and last segment measured along the filament, the total number of cells measured, the mean and standard deviation of ^{13}C atom fractions of all measured cells within a filament, the mean and standard deviation of the assimilation rate constant and the corresponding coefficient of variation within that filament. Data show that variability within filaments is clearly lower than variability among filaments (compare with **Table S1**).

Filament	Max length (mm)	No. cells	No. cells measured	$^{13}C/(^{12}C+^{13}C)$	k_{DIC} (d^{-1})	Coefficient of variation
red	2.323	774	63	0.078 ± 0.0036	0.910 ± 0.081	8.9
green	1.570	523	65	0.069 ± 0.0043	0.736 ± 0.081	11.0
yellow	1.431	477	59	0.081 ± 0.0061	1.001 ± 0.153	15.3
blue	1.555	518	42	0.034 ± 0.0023	0.229 ± 0.025	11.1
purple	0.984	328	23	0.022 ± 0.0011	0.106 ± 0.011	10.0
white	0.797	266	23	0.022 ± 0.0011	0.103 ± 0.011	10.4
black	0.790	263	16	0.061 ± 0.0071	0.596 ± 0.114	19.2
cyan	1.002	334	34	0.019 ± 0.0013	0.072 ± 0.013	18.7

Movie S1 (separate file). Time-lapse taken with a phase contrast microscope showing the sudden change in the direction of motion of cable bacteria. Two types of morphologically different cable bacteria are seen; very thin cable bacteria and cable bacteria with a larger diameter. The bottom cable bacterium with a large diameter shows a sudden reversal of movement even though the oxygen front was stable. Note that within the sediment enrichment used for nanoSIMS only the cable bacteria with a large diameter were present (diameter ~3-4 µm).

Dataset 1 (separate file). Document with each ^{13}C atom fraction, ^{15}N atom fraction and $^{31}P/C$ value for each filament segment. These values were used to construct Fig. 1, SI Appendix table S1 and Fig. S2.

Dataset 2 (separate file). Document with each ^{13}C atom fraction for each single cell from the each followed filaments. These values were used construct Fig. 2 and SI Appendix table S2.

SI References

1. Malkin SY, et al. (2014) Natural occurrence of microbial sulphur oxidation by long-range electron transport in the seafloor. *ISME J* 8(9):1843–1854.
2. Sulu-Gambari F, et al. (2016) Cable Bacteria Control Iron-Phosphorus Dynamics in Sediments of a Coastal Hypoxic Basin. *Environ Sci Technol* 50(3):1227–1233.
3. Nielsen LP, Risgaard-Petersen N, Fossing H, Christensen PB, Sayama M (2010) Electric currents couple spatially separated biogeochemical processes in marine sediment. *Nature* 463(7284):1071–1074.
4. Meysman FJR, Risgaard-Petersen N, Malkin SY, Nielsen LP (2015) The geochemical fingerprint of microbial long-distance electron transport in the seafloor. *Geochim Cosmochim Acta* 152:122–142.
5. Vasquez-Cardenas D, et al. (2015) Microbial carbon metabolism associated with electrogenic sulphur oxidation in coastal sediments. *ISME J*:1–13.
6. Schauer R, et al. (2014) Succession of cable bacteria and electric currents in marine sediment. *ISME J*:1–9.
7. Polerecky L, et al. (2012) Look@NanoSIMS - a tool for the analysis of nanoSIMS data in environmental microbiology. *Environ Microbiol* 14(4):1009–1023.
8. Coplen TB (2011) Guidelines and recommended terms for expression of stable-isotope-ratio and gas-ratio measurement results. *Rapid Commun Mass Spectrom* 25(17):2538–2560.
9. Li Z-P, et al. (2007) Determination of isotope composition of dissolved inorganic carbon by gas-chromatography-conventional isotope-ratio mass spectrometry. *Chinese J Anal Chem* 35(10):1455–1458.
10. Solorzano I (1969) Determination of ammonia in natural seawater by the phenol-hypochlorite method. *Limnol Oceanogr* 14(5):799–801.
11. Pfeffer C, et al. (2012) Filamentous bacteria transport electrons over centimetre distances. *Nature* (491):10–13.
12. Nielsen LP, Risgaard-Petersen N (2015) Rethinking Sediment Biogeochemistry After the Discovery of Electric Currents. *Ann Rev Mar Sci* 7(1):425–442.
13. Damgaard LR, Risgaard-Petersen N, Nielsen LP (2014) Electric potential microelectrode for studies of electrobiogeophysics. *J Geophys Res Biogeosciences* 119(9):1906–1917.
14. Bjerg JT, Damgaard LR, Holm SA, Schramm A, Nielsen LP (2016) Motility of electric cable bacteria. *Appl Environ Microbiol* 82(13):3816–3821.
15. Dinno A (2017) Package ‘dunn.test.’ *CRAN Repos*:1–7.
